# Mutilación genital femenina: Reflexiones desde el terreno para una formación sanitaria intercultural

**DOI:** 10.23938/ASSN.1140

**Published:** 2025-10-16

**Authors:** Francisco Miguel González Valverde

**Affiliations:** Servicio de Cirugía General y del Aparato Digestivo Hospital General Universitario Reina Sofía de Murcia Murcia España; Departamento de Cirugía, Pediatria, Ginecologia y Obstericia Universidad de Murcia Murcia España

## Sr. Editor:

Como cirujano y miembro de una ONG sanitaria, he tenido la oportunidad de trabajar en países como Senegal, Mauritania, Mali y Kenia durante casi tres décadas. En esta situación, he atendido a numerosas mujeres víctimas de mutilación genital femenina, muchas de ellas en condiciones de salud muy precarias.

He leído atentamente el artículo *“Validación de un cuestionario sobre conocimientos y actitudes ante la mutilación genital femenina y su abordaje sanitario en estudiantes de enfermería: Estudio piloto”* de María del Mar Pastor Bravo y col^1^, publicado en el número 48(2) de la revista *Anales del Sistema Sanitario de Navarra*. Tras su lectura, deseo compartir algunas reflexiones, derivadas de mi experiencia sobre el terreno, que podrían complementar la perspectiva académica y sanitaria española con una visión también práctica y culturalmente situada.

En primer lugar, celebro la iniciativa de validar un instrumento específico para evaluar el conocimiento, las actitudes y el abordaje clínico de la mutilación genital femenina en estudiantes de enfermería. Sin embargo, ignoro si han tenido en cuenta la inclusión de la perspectiva intercultural y del conocimiento práctico adquirido por profesionales que trabajan en países donde la mutilación genital femenina sigue siendo una práctica vigente, legal o tolerada socialmente. Por ejemplo, en Guinea y Mali he constatado cómo la tipología de la mutilación genital femenina (clasificación OMS I-IV) a menudo no se corresponde con una realidad clínica nítida. En muchas ocasiones, se observan formas mixtas o mal clasificadas de mutilación, realizadas por personas sin formación sanitaria alguna y con técnicas rudimentarias. Esto complica no solo su identificación sino también su reconstrucción quirúrgica, una práctica cada vez más demandada por mujeres jóvenes que desean revertir, en la medida de lo posible, las consecuencias físicas y psicológicas de la mutilación genital femenina^2^^,^^3^.

En segundo lugar, el artículo señala con acierto la importancia del abordaje desde la atención primaria en España. No obstante, quisiera subrayar la necesidad de preparar también al personal de urgencias hospitalarias, cirugía general, y ginecología y obstetricia en la atención de las complicaciones derivadas de la mutilación genital femenina, como fístulas, quistes, infecciones recurrentes o partos distócicos^4^^,^^5^. En situaciones menos graves, la intervención quirúrgica puede enfocarse en corregir los daños visibles y mejorar la apariencia estética de los genitales. En casos más complejos, pueden requerirse procedimientos reconstructivos avanzados que incluyan injertos de piel y técnicas microquirúrgicas para recuperar tanto la funcionalidad como la sensibilidad. La formación quirúrgica especializada, incluso en contextos precarios, debe ser parte de un enfoque integral, ya que estas cirugías no solo mejoran la salud física, sino también la autoestima de las mujeres afectadas.

Por otro lado, el cuestionario validado en el estudio, aunque útil para medir conocimientos básicos, no parece incorporar aspectos vinculados al contexto legal y cultural de los países de origen de estas mujeres, donde la mutilación se considera un rito de paso hacia la vida adulta y un prerrequisito matrimonial. ¿Qué ocurre cuando una mujer expresa libremente su deseo de practicar la mutilación genital femenina en su hija, ya sea por presión familiar o por temor al rechazo social? ¿Está preparado el personal sanitario español para intervenir en esos casos sin imponer una visión punitiva, pero garantizando los derechos de la menor? En cualquier contexto, la inclusión del entorno familiar en la toma de decisiones ofrece mayores garantías de adhesión a los procesos de cambio.

En Senegal o Kenia, por ejemplo, trabajamos con líderes religiosos y comunitarios que apoyan activamente campañas contra la mutilación genital femenina, pero solo cuando se les involucra desde el respeto y no desde el juicio externo. Algunos grupos se oponen a su prohibición por temor a que las niñas que no se sometan al procedimiento no consigan casarse o sean estigmatizadas como promiscuas. Esta experiencia me lleva a respaldar plenamente su propuesta de que la educación sanitaria sobre mutilación genital femenina en España no se limite a protocolos, legislación y guías clínicas, sino que incluya formación en mediación cultural, escucha activa y construcción de confianza con las comunidades migrantes^6^^,^^7^.

Por último, deseo señalar que las víctimas de mutilación genital femenina no deben ser vistas únicamente como pacientes, sino también como posibles agentes de cambio. En algunas regiones de África Occidental, como Costa de Marfil, colaboramos con mujeres mutiladas que actúan como educadoras sanitarias en sus propias comunidades, promoviendo alternativas culturales no violentas y facilitando el abandono de la práctica^8^^,^^9^.

El estudio presentado por María del Mar Pastor Bravo y col^1^ representa un avance necesario, pero me parece importante complementar esta aproximación cuantitativa con vivencias cualitativas y testimonios clínicos de mujeres mutiladas y de profesionales que hayan trabajado directamente en países donde la mutilación genital femenina no es un concepto académico, sino una realidad cotidiana. La lucha contra esta forma de violencia requiere ciencia, sí, pero también escucha, humildad y cooperación intercultural.

Enhorabuena por su estudio.

## Data Availability

Se encuentran disponibles bajo petición al autor de correspondencia.

## References

[1] Pastor Bravo MM, Martínez Jurado YM, Ayad Ouchen S (2025). Validación de un cuestionario sobre conocimientos y actitudes ante la mutilación genital femenina y su abordaje sanitario en estudiantes de enfermería: Estudio piloto. An Sist Sanit Navar.

[2] González-Timoneda A, Cano-Sánchez A, Ruiz Ros V (2021). Female genital mutilation consequences and healthcare received among migrant women: A phenomenological qualitative study. Int J Environ Res Public Health.

[3] Buggio L, Facchin F, Chiappa L, Barbara G, Vercellini P (2019). Psychosexual consequences of female genital mutilation and the impact of reconstructive surgery: A narrative review. Health Equity.

[4] World Health Organization (2018). Care of girls and women living with female genital mutilation: A clinical handbook.

[5] Almroth L, Almroth-Berggren V, Hassanein OM, Al-Said SS, Hasan SS, Lithell UB (2001). Male complications of female genital mutilation. Soc Sci Med.

[6] UNAF (2020). Guía para abordar la mutilación genital femenina en las entrevistas con niñas y/o sus familias.

[7] Sequeira-Aymar E, DiLollo X, Osorio-López Y, Gonçalves AQ, Subirà C, Requena-Méndez A (2019). Recomendaciones para el cribado de enfermedad infecciosa, salud mental y mutilación genital femenina en pacientes inmigrantes atendidos en Atención Primaria. Aten Primaria.

[8] Shell-Duncan B, Wander K, Hernlund Y, Moreau A (2013). Legislating change? Responses to criminalizing female genital cutting in Senegal. Law Soc Rev.

[9] UNICEF (2016). Female Genital Mutilation/Cutting: A Global Concern.

[10] Kaplan Marcusán A, Ajenjo Cosp M, López Gay A (2022). Mapa de la mutilación genital femenina en España 2021.

